# Health Tract, No. 258

**Published:** 1866-11

**Authors:** 


### COURTING.

In the old world marriage is a matter of convenience, or an out and out business transaction; and family is bartered for funds, or an improvement in the pecuniary affairs of both parties is aimed at. In our own country it is literally a "love affair," without rhyme or reason, sense or system;—it is a blissful, mutual absorbtion of two hearts into one — for awhile any how. Perhaps if it were made a matter of Hygiene there would be eventually a greater amount of happiness and solid prosperity in any community. A sickly wife has many a time blasted the ambition of an industrious and enterprising young man whose aim was to rise in his business and become one of the leading men of his calling.

But in the very first year sickness came, the young wife could not attend to her domestic affairs; the servants became remiss, indifferent and wasteful; the physician was called in; the husband himself was obliged to remain at the house and the same derangement of his own affairs took place, and every where there was waste and expenditure and loss of business and custom.—Discouragement came, until finally all that was hoped for was to live from one day to another.

At other times the husband became the invalid; the support of the family is thrown upon the wife and the mother; and how many of them have worked themselves into a premature grave or into a lunatic asylum, it is painful te contemplate.

No sickly person can honorably marry another in good health without previously making a fair statement of the case. And even then if a marriage takes place a crime has been committed against the community and against unborn innocents. But when both the parties are "sickly" it is wholly inexcusable, and ought to be frowned upon by every intelligent community, however satisfactory the pecuniary condition of the parties. They may be able to support themselves but they can give no guarantee that their children, diseased in body and feeble in mind, shall not be a public charge at the hospital, the poor-house or an insane-asylum. The best general plan for ensuring a healthy and vigorous offspring is to make an antipodal marriage; — to make as much of a cross in the physical characteristics as possible. The city should marry the country; the black-haired the blond; the bilious temperament the nervous; the fair-skinned the brunette; the stout the slender; the tall the short. To marry each its like, is to degrade the race.

